# Digital Clock Drawing as an Alzheimer’s Disease Susceptibility Biomarker: Associations with Genetic Risk Score and APOE in Older Adults

**DOI:** 10.14283/jpad.2023.48

**Published:** 2023-04-28

**Authors:** Louisa I. Thompson, M. Cummings, S. Emrani, D. J. Libon, A. Ang, C. Karjadi, R. Au, C. Liu

**Affiliations:** 1 Dept. of Psychiatry & Human Behavior, Alpert Medical School, Brown University, 345 Blackstone Blvd., Providence, RI 02906 USA; 2 Dept. of Anatomy & Neurobiology, Boston University Medical School, Boston, MA USA; 3 Dept. of Geriatrics, Gerontology, and Psychology, New Jersey Institute for Successful Aging, School of Osteopathic Medicine, Rowan University, Glassboro, New Jersey USA; 4 Framingham Heart Study, Boston University Medical School, Boston, MA USA; 5 Dept. of Biostatistics, Boston University School of Public Health, Boston, MA USA

**Keywords:** Genetics, polygenic risk score, APOE, cognitive screening, clock drawing test, digital biomarkers

## Abstract

**Background:**

Alzheimer’s disease (AD) is the leading cause of dementia in older adults, but most people are not diagnosed until significant neuronal loss has likely occurred along with a decline in cognition. Non-invasive and cost-effective digital biomarkers for AD have the potential to improve early detection.

**Objective:**

We examined the validity of DCTclock™ (a digitized clock drawing task) as an AD susceptibility biomarker.

**Design:**

We used two primary independent variables, Apolipoprotein E (APOE) ε4 allele carrier status and polygenic risk score (PRS). We examined APOE and PRS associations with DCTclock™ composite scores as dependent measures.

**Setting:**

We used existing data from the Framingham Heart Study (FHS), a community-based study with the largest dataset of digital clock drawing data to date.

**Participants:**

The sample consisted of 2,398 older adults ages 60–94 with DCTclock™ data (mean age of 72.3, 55% female and 92% White).

**Measurements:**

PRS was calculated using 38 variants identified in a recent large genome-wide association study (GWAS) and meta-analysis of late-onset AD (LOAD).

**Results:**

Results showed that DCTclock™ performance decreased with advancing age, lower education, and the presence of one or more copies of APOE ε4. Lower DCTclock™ Total Score as well as lower composite scores for Information Processing Speed (both command & copy conditions) and Drawing Efficiency (command condition) were significantly associated with higher PRS levels and more copies of APOE ε4. APOE and PRS associations displayed similar effect sizes in both men and women.

**Conclusions:**

Our results indicate that higher AD genetic risk is associated with poorer DCTclock™ performance in older adults without dementia. This is the first study to demonstrate significant differences in clock drawing performance on the basis of APOE status or PRS.

**Electronic Supplementary Material:**

Supplementary material is available in the online version of this article at 10.14283/jpad.2023.48.

## Introduction

Alzheimer’s disease (AD) is a neurodegenerative disease projected to affect 12.7 million adults age 65 and older by 2050 (1). Despite this growing prevalence, AD pathophysiology remains difficult to detect for clinical diagnostic purposes. Biomarkers from cerebrospinal fluid (CSF) and advanced neuroimaging technology, such as amyloid beta (Aβ) positron emission tomography (PET) scans, are invasive and costly to obtain and are currently accessible in a limited capacity (e.g., in research trials or specialty clinics). Recent research and development suggests that several novel, minimally invasive digital and biological biomarkers (e.g., genetic and blood-based) have the potential to yield diagnostic value similar to PET and CSF measures, especially when combined (2, 3). Surrogate neuropsychological biomarkers may be particularly beneficial for identifying individuals at risk for emergent AD in primary care and for secondary prevention AD clinical drug trials while significantly reducing patient burden (4–6).

In older adults with superficially intact cognition, digital neuropsychological biomarkers gleaned using automated and advanced analytics may provide information about subtle variations in cognition (e.g., response latency, decision making, graphomotor output) associated with AD neuropathology and brain function in areas implicated in AD pathogenesis (7, 8). Many of these nuanced behaviors cannot be easily captured using traditional pencil-paper tests or are infeasible for clinicians to record and interpret reliably (9, 10).

DCTclock™ is one of a growing number of digitally administered neuropsychological tests that may be useful for obtaining digital cognitive biomarkers for AD and related dementias. Based on the traditional clock drawing task commonly used for dementia screening (11, 12) DCTclock™ uses a digital pen to capture a person’s drawings of a clock to command (drawing a de novo clock) and copy (copying a model clock) using standard clock drawing instructions. Over the past several decades, digital clock drawing test (dCDT) research using raw digital pen-generated data has demonstrated that machine-learning algorithms using features extracted from the dCDT can predict neuroimaging biomarkers of AD and can distinguish between normal cognition, mild cognitive impairment, and dementia (13–18). Building on this prior work, DCTclock™ utilizes a machine learning-derived scoring algorithm, provides a cloud-based scoring platform, and generates four age-adjusted composite scores for both command and copy test conditions, as well as a composite total command/copy score designed to aid clinical interpretation. DCTclock™ is FDA approved for clinical use and is commercially available as part of the Linus Health platform.

The extant literature on DCTclock™ has reported associations between DCTclock™ performance and Aβ burden in the frontoparietal region and default mode networks in normal individuals (16). DCTclock™ summary score and spatial reasoning composite scores were associated with greater Aβ and tau burden (16). The composite scores have also been shown to distinguish accurately between normal cognition and mild cognitive impairment (MCI), including amnestic and dysexecutive MCI profiles (19). DCTclock™ may therefore be a potential tool that could efficiently address the need for surrogate biomarkers detecting early AD processes. However, DCTclock™ composite measures have yet to be independently validated in a community-based sample of older adults.

In addition to digital biomarkers, genetic biomarkers are another important indicator of risk for AD. The heritability of late onset Alzheimer’s disease (LOAD) is estimated to be 60–80%, with Apolipoprotein E (APOE) ε4 allelic variants contributing the largest effect (20–22). APOE ε4 carriers are at higher risk for AD and tend to accumulate Aβ at an earlier age and have greater tau accumulation in the medial temporal lobe (22, 23). APOE ε4 confers a stronger dementia risk in women compared to men, and is associated with earlier dementia onset in women (22). AD risk factors are also discovered through polygenic risk score (PRS) analysis using effect size estimates attained from established genomic-wide association studies (GWAS) (24). PRS has been shown to discriminate between controls and confirmed cases with AD at up to 84% accuracy and can be calculated at any point in an individual’s life (25). Further, PRS predicts longitudinal cognitive decline in cognitively normal individuals with and without high tau and Aβ burden at autopsy, and among APOE ε4 non-carriers (26, 27). PRS is associated with cognitive decline in areas of processing speed and working memory in cognitively normal individuals (26), and episodic verbal memory in pre-clinical AD (28). There also appears to be considerable sex-dependent differences in the genetic architecture and therefore, polygenic risk in AD (29–31). Comparatively, a large study sample of twins found that genetic etiology of AD may be similar between women and men (32). Results regarding non-APOE ε4 genetic risk across sexes remains inconclusive, and may be modulated by sex-specific biological factors (e.g. hormones) and psychosocial risk.

The current study seeks to contribute to the validation of DCTclock™ performance as a potential AD susceptibility biomarker for use in AD clinical trials and other AD risk screening efforts (e.g., primary care). We examined DCTclock™ composite and total scores generated from dCDT collected in the Framingham Heart Study (FHS), a community-based study with largest dataset of raw dCDT data to date. Using a GWAS available for FHS participants, we examined associations between DCTclock™ performance and the genetic biomarkers of APOE ε4 alone and a PRS containing APOE ε4. In order to validate our potential digital biomarkers against known genetic biomarkers.

## Methods

### Study population

This study consisted of multiple generations of participants in the Framingham Heart Study (FHS). The original cohort (GEN 1) recruited 5,209 residents of Framingham, Massachusetts to investigate cardiovascular disease (CVD) epidemiology and risk factors (33). In 1971, the FHS recruited 5,214 new participants, who were biological children of the original cohort and the spouses of those children, in the Offspring (GEN 2) cohort (34, 35). Beginning in 2002, a third generation of 4,095 participants (GEN 3), who were grandchildren of Gen 1, was recruited for continued study of CVD. Also in 2002, the New Offspring Spouse (NOS) cohort of 103 individuals was enrolled. NOS included parents of GEN 3 participants who had not yet been enrolled in FHS (36). In 1994, reflecting the growing ethnic diversity of Framingham and the surrounding area, the FHS enrolled 506 individuals of Hispanic, non-Hispanic black, Asian, and Native-American descent as the Omni 1 cohort. In 2003, the Omni 2 cohort of 410 ethnically diverse participants was enrolled, some of whom were family members of the Omni 1 participants (37, 38). The FHS has collected longitudinal measures such as demographics, cardiovascular risk factors, biomarker data, co-morbidities and incident disease including CVD and AD, through regular health exams and ancillary studies (38, 39). Dementia diagnosis is made via diagnostic case conference. The methods of FHS dementia surveillance and diagnosis have been previously described (40). The Boston University Medical Campus Institutional Review Board approved protocols for participant examinations and collection of genetic materials were approved. Written informed consent for genetic studies was obtained for all participants.

### Digital clock measurements

Traditional (paper and pencil) clock drawing assessments are well established in neuropsychological research to characterize participants with neurodegenerative disorders including AD (41–43). The digital clock drawing test (dCDT) has been integrated into the FHS neuropsychological testing protocol since 2011 using an algorithm created by the MIT/Lahey lab. In addition to continuing collection with that algorithm, the use of the DCTclock™ algorithm, created and owned by Linus Health, began in 2018 (16). The baseline dCDT results that were first collected for each participant are being used. Participants are presented with a paper test form containing a faint dot pattern and handed a digital pen that looks and functions like a normal pen but contains a camera sensor that captures pen position every 12ms by tracking the pen’s position relative to the dots on the paper. The instructions of the dCDT are consistent with traditional CDT administration and include both command and copy test conditions. In the command condition, participants are asked to “draw the face of a clock, put in all numbers, and set the hands to 10 after 11.” Upon completion of the command test condition, the copy test condition is administered whereby participants are asked to copy a model of a clock with hands set to ‘10 after 11’. The digital pen allows for the capture of thousands of clock drawing features to be analyzed as a series of time-stamped (x,y) coordinates. DCTclock™ machine learning algorithms were developed to calculate meaningful clock scores based on their ability to discriminate performance between thousands of healthy controls and participants from different diagnostic groups, including amnestic MCI, AD dementia, Parkinson’s disease, and other neurodegenerative disorders (14, 44). Details on the DCTclock™ algorithm and scoring process have been described in detail elsewhere (16). Supplemental Table 1 contains a description of the nine DCTclock™ indices used for this analysis, which include a total composite score and command (COM) and copy (COP) condition composite scores for Information Processing, Drawing Efficiency, Spatial Reasoning, and Simple/Complex Motor Operations. These domain-specific composite scores are constructed by DCTclock™ by combining machine learning calculations of variables that are related to each domain (16).

**Table 1:** Characteristics of the Framingham Heart Study sample

**Participant characteristics:**	**Total Sample (N = 2398)**	**Men (n =1071, 46.0%)**	**Women (n = 1327, 54.0%)**
Non-White n (%)	198 (8.26%)	79 (7.38%)	119 (8.97%)
Age, mean (sd)	72.3 (7.86)	72.1 (7.88)	72.4 (7.84)
Dementia, n (%)	63 (2.63%)	32 (2.99%)	31 (2.34%)
APOE, n (%)*	482 (20.1%)	224 (20.9%)	258 (19.4%)
PRS, mean (sd)	0.298 (0.0338)	0.297 (0.0341)	0.299 (0.0335)
Education, n (%)			
No high school	58 (2.46%)	31 (2.96%)	27 (20.7%)
High school	451 (19.2%)	160 (15.3%)	291 (22.2%)
Some college	662 (28.1%)	241 (23.0%)	421 (32.3%)
College graduate	1183 (50.3%)	617 (58.8%)	566 (43.4%)
DCTclock™ Scores: median (interquartile range)			
DCT Total Score	79.0 (30.5)	77.1 (30.8)	81.0 (31.3)
COM Drawing Efficiency	63.3 (12.6)	63.0 (12.6)	63.6 (12.5)
COM Simple Motor	63.6 (11.3)	62.7 (12.3)	64.6 (10.1)
COM Information Processing	61.7 (13.4)	61.9 (13.2)	61.5 (13.7)
COM Spatial Reasoning	69.2 (23.9)	68.9 (23.4)	69.4 (24.2)
COP Drawing Efficiency	62.4 (9.96)	62.0 (9.56)	62.7 (10.4)
COP Simple Motor	60.8 (8.81)	60.3 (9.71)	61.1 (7.92)
COP Information Processing	62.2 (15.5)	61.5 (15.0)	63.1 (15.5)
COP Spatial Reasoning	70.9 (26.3)	70.9 (26.6)	71.0 (25.4)

*Individuals with APOE ε3ε4 or ε4ε4 genotypes

### Genotyping and imputation

Genotyping was performed using Affymetrix 500 K and MIPS 50 K platforms (45, 46) in the FHS. The quality control procedures on the genotyping data were previously described in detail (47). In brief, single nucleotide polymorphisms (SNPs) were removed if Hardy–Weinberg Equilibrium (HWE) P values were below 1E-6. SNPs were also removed for call rates below 96.9%, minor allele frequencies (MAF) below 0.01, map mismatches between Build 36 and Build 37, and not having a physical location or duplication or having Mendelian error number below 1000 (47). MaCH software (48) was used for imputation in conjunction with the 1000G phase 3 version 5 to generate an imputed set of ∼30 million variants (49). Single nucleotide polymorphisms (SNPs) with imputation quality ratio R-squared <0.5 or MAFs <0.01 were removed.

### APOE genotype and polygenic risk score construction

We used two primary independent variables of interest in association analyses with the DCTclock™ composite measures: APOE carrier status and polygenic risk score (PRS). The APOE genotype manifests as three major alleles, ε2, ε3 and ε4 and the presence of APOE ε4 is the strongest genetic risk factor for AD (50, 51). Therefore, we classified the participants into two groups by the presence (ε3/ε4 and ε4/ε4) or absence (ε2/ε3, ε2/ε2 and ε3/ε3) of the ε4 allele. To avoid a mixed allele effect, we also excluded participants with ε2/ε4 (n = 203) despite the presence of the ε4 allele because APOE ε2 has a protective effect against developing AD (52). The APOE genotype was determined directly as reported previously (53).

A recent large genome-wide association study (GWAS) and meta-analysis was conducted on late-onset Alzheimer’s disease (LOAD) in participants 65 years and above (54). This study analyzed 90,338 cases with LOAD and 1,036,225 controls and identified 38 variants, seven of which were novel. Thus, these 38 variants were used to develop the PRS for this investigation. We firstly harmonized the effect (or coded) alleles between this study and the FHS imputation data so that the effect size (β_i) reflected the effect of a risk allele of a SNP for higher odds for LOAD. We then multiplied the effect size of an individual variant and allele dosages of this variant for all FHS participants. The weighted genetic risk score for a participant was then constructed by the summation of the weighted dosage across the 38 variants. This process is summarized in the formula below:

$$PRS=\sum\nolimits_{1}^{38}\beta_{i}\ast dosage$$

### Covariates

Covariates included self-reported sex, a continuous variable of age at the time of DCTclock™ completion, and categorical level of education grouped (i.e., less than high school completion, high school graduate, some college, and college graduate).

### Association analysis between PRS/APOE and composite digital clock markers

We first performed univariate analyses to quantify the relationships of composite digital clock markers with age, sex, and education. We then applied linear mixed models to investigate associations of APOE or PRS as the predictor variable with each of the composite digital clock markers as an outcome variable. In addition, family structure was adjusted in linear mixed models (55). The association analyses were performed in all participants, in men-only and women-only samples. All statistical analyses were performed using R software (version 4.2.2). The “nlme” library was used for linear mixed modeling (56). We used two-sided p ≤ 0.05 for significance.

## Results

### Participant characteristics

The source data for this study consisted of 2,398 FHS participants and their DCTclock™ results. Although our analyses originally were performed on a larger sample of 3,908 participants ranging from ages 27–99, we learned that this sample exceeded the lower and upper age limits for which the DCTclock™ scoring algorithm was developed. Subsequently, the sample was restricted to individuals who were ages 60–94 at the time they completed the clock drawing task (Supplemental Table 2), consistent with the age range of validated use for the DCTclock™. This dataset included more women (n = 1327, 55.3%) than men (n = 1071, 44.7%) (Table 1). Women were slightly older than men, with a mean age 72.4 (range 60 to 94) in women versus 72.1 in men (range 60 to 94). The majority of the participants were of White European descent, with 79 men (7.38%) and 119 women (8.97%) identifying as non-White. In general, men had more education than women: 58.8% of men and 43.4% of women were college graduates, while 38.3% of men and 54.5% of women had only high school or some college education. In total, 63 participants (2.63%) had been diagnosed with prevalent dementia, consisting of 32 men (2.99%) and 31 women (2.34%). We removed participants who had missing values in any of the covariates or in outcome variables. The association analyses were performed in up to 2,235 participants with APOE ε4 carrier status as the predictor variable and in up to 2,033 participants with PRS as the predictor variable.

### Association analyses of DCTclock™ with APOE and PRS

#### Demographics

We investigated the association of age, sex, and education with performance in DCTclock™ composite measures. We found that performance in all composite DCTclock™ measures decreased with advancing age and increased with higher education levels (Supplemental Tables 3–5). For example, for each year of increase in age, on average, DCTclock™ Total Score was 1.16 units lower (p < 0.001). A higher education level, on average, was associated with 5.37 units higher score in the DCTclock™ Total Score (p < 0.001). Women performed better than men across most composite measures, with significant differences seen for Total Score, Information Processing, and Simple Motor scores. For example, women, on average, had about 2.39 units higher DCTclock™ Total Score than men (p = 0.014).

#### Associations with APOE ε4 status

As expected, on average, carrying at least one APOE ε4 allele was associated with lower composite scores after adjusting for age, sex, and education level (Table 2). APOE carrier status was significantly associated with the DCTclock™ Total Score (a single score between 0 and 100 that captures overall performance across command and copy conditions). Specifically, participants with at least one APOE ε4 allele had a 2.59-unit lower DCTclock™ Total Score (p = 0.023) compared to those without an APOE ε4 allele. Participants carrying at least one APOE ε4 allele also displayed lower COP Information Processing scores (efficiency the participant demonstrated during the process of drawing the copy clock) (beta = 0.61, p = 0.020) and a trend of lower COP Spatial Reasoning scores (spatial abilities demonstrated during the process of drawing the copy clock) (beta = 0.99, p = 0.064).

**Table 2:** Association analyses of APOE/PRS with nine DCTclock™ composite scores

**Full association**								
					**PRS**			
**Variable**	**n**	**Beta**	**SE**	**p-value**	**n**	**Beta**	**SE**	**p-value**
DCT Total Score	2208	−2.59	1.14	0.023	2011	−28.9	14	0.040
COM Drawing Efficiency	2235	−0.97	0.6	0.107	2033	−24.9	7.32	< 0.001
COM Simple Motor	2219	−0.21	0.46	0.645	2020	−9.41	5.74	0.101
COM Information Processing	2235	−0.55	0.54	0.314	2033	−18.3	6.79	0.007
COM Spatial Reasoning	2224	−1.68	1.01	0.097	2025	−3.25	12.4	0.792
COP Drawing Efficiency	2230	−0.6	0.49	0.219	2030	−10.4	5.99	0.084
COP Simple Motor	2224	−0.15	0.36	0.689	2026	−0.9	4.58	0.843
COP Information Processing	2230	−1.42	0.61	0.020	2030	−14.8	7.57	0.051
COP Spatial Reasoning	2224	−1.83	0.99	0.064	2026	−10.3	12.5	0.407

Linear Mixed Model; (DCT marker) ∼ APOE/PRS + age + sex + education

#### Associations with Polygenic Risk Score

The PRS was significantly associated with the DCTclock™ Total Score (beta = −28.9, p = 0.040). One unit increase in PRS was significantly associated with a 28.9 unit decrease in DCTclock™ Total Score. A higher level of PRS was also significantly associated with poorer performance in COM Drawing Efficiency (The efficiency the participant demonstrated during the process of drawing the command clock) (beta = −24.9, p < 0.001), COM Information Processing (beta = −18.3, p = 0.007), and a trend of lower COP Information Processing (efficiency the participant demonstrated during the process of drawing the copy clock) (beta = −14.8, p = 0.051).

#### Sex-stratified associations

Sex is an important biological variable. As secondary analyses, we performed sex-specific analyses to investigate whether the associations of DCTclock™ measures with APOE/PRS were different in men and women. (Table 3 and Table 4). As noted above, women appear to have better performance on DCTclock™ measures compared to men. We found significant associations in men between APOE carrier status and both COM Spatial Reasoning (beta = −3.29, p = 0.028) and COP Information Processing (beta = −1.68, p = 0.050). We also found significant associations in men between the PRS and COM Drawing Efficiency (beta = −30.0, p = 0.008) and COM Information Processing (beta = −26.7, p = 0.008). We observed significant associations in women between APOE carrier status and both DCTclock™ Total Score (beta = −3.31, p = 0.033) and COP Spatial Reasoning (beta = −3.07, p = 0.021). We also observed significant associations in women between the PRS and COM Drawing Efficiency (beta = −21.9, p = 0.024), COM Simple Motor (beta = −18.7, p = 0.008), and COP Drawing Efficiency (beta = −19.9, p = 0.018). However, the difference in association effects between dCDT measures and APOE carrier status or PRS were not statistically significant, with all p-values above 0.1.

**Table 3:** Association analyses of APOE/PRS with nine DCTclock™ composite scores, men only

					**PRS**			
**Variable**	**n**	**Beta**	**SE**	**p-value**	**n**	**Beta**	**SE**	**p-value**
DCT Total Score	988	−2.62	1.7	0.123	902	−30.3	21.9	0.167
COM Drawing Efficiency	1001	−1.5	0.9	0.095	913	−30	11.3	0.008
COM Simple Motor	992	−0.1	0.73	0.887	905	0.16	9.46	0.987
COM Information Processing	1001	−0.76	0.78	0.336	913	−26.7	10	0.008
COM Spatial Reasoning	995	−3.29	1.5	0.028	908	−1.37	18.9	0.942
COP Drawing Efficiency	1001	−0.27	0.68	0.691	914	−3.63	8.63	0.674
COP Simple Motor	997	0.15	0.58	0.803	910	8.13	7.65	0.289
COP Information Processing	1001	−1.68	0.86	0.050	914	−11.7	10.8	0.279
COP Spatial Reasoning	997	−0.39	1.49	0.792	910	−18.1	19.4	0.352

Linear Mixed Model; (DCT marker) ∼ APOE/PRS + age + sex + education

**Table 4:** Association analyses of APOE/PRS with nine DCTclock™ composite scores, women only

**Women**								
						**PRS**		
**Variable**	**n**	**Beta**	**SE**	**p-value**	**n**	**Beta**	**SE**	**p-value**
DCT Total Score	1220	−3.31	1.54	0.033	1109	−32.9	18.5	0.076
COM Drawing Efficiency	1234	−0.66	0.81	0.413	1120	−21.9	9.65	0.024
COM Simple Motor	1227	−0.34	0.58	0.559	1115	−18.7	7.07	0.008
COM Information Processing	1234	−0.59	0.75	0.437	1120	−11.7	9.31	0.210
COM Spatial Reasoning	1229	−0.69	1.36	0.612	1117	−3.68	16.3	0.821
COP Drawing Efficiency	1229	−1.1	0.69	0.110	1116	−19.9	8.35	0.018
COP Simple Motor	1227	−0.36	0.45	0.422	1116	−8.5	5.52	0.124
COP Information Processing	1229	−1.49	0.86	0.085	1116	−19.4	10.6	0.066
COP Spatial Reasoning	1227	−3.07	1.33	0.021	1116	−1.94	16.6	0.907

Linear Mixed Model; (DCT marker) ∼ APOE/PRS + age + sex + education

#### Association analyses in participants without dementia

Some FHS participants had been diagnosed with dementia when DCTclock™ measures were conducted, which might have had an effect on the results by skewing the results towards worse DCTclock™ performance. In order to examine the potential of DCTclock™ performance as a digital biomarker of AD susceptibility, we performed analyses where prevalent cases of dementia were removed (n = 63; Table 5). Results in participants without dementia were similar to those in the full sample. However, the association strength was attenuated. The associations between PRS and both COM Drawing Efficiency (beta = −24.2, p < 0.001) and COM Information Processing (beta = −24.2, p = 0.010) persisted. However, there were no longer any significant associations with APOE carrier status.

**Table 5:** Association analyses of APOE/PRS with nine digital cognition component scores, with 63 individuals with diagnosed dementia removed

**Dementia removed**								
	**APOE**				**PRS**			
**Variable**	**n**	**Beta**	**SE**	**p-value**	**n**	**Beta**	**SE**	**p-value**
DCT Total Score	2159	−1.78	1.12	0.113	1964	−23.2	13.6	0.089
COM Drawing Efficiency	2177	−0.84	0.58	0.148	1979	−24.2	7.06	< 0.001
COM Simple Motor	2167	−0.09	0.46	0.846	1970	−8.32	5.66	0.142
COM Information Processing	2177	−0.24	0.53	0.654	1979	−17	6.62	0.010
COM Spatial Reasoning	2171	−1.16	1	0.246	1974	4.51	12.1	0.709
COP Drawing Efficiency	2172	−0.15	0.46	0.738	1976	−8.52	5.59	0.128
COP Simple Motor	2169	0.08	0.36	0.832	1973	0.52	4.56	0.909
COP Information Processing	2172	−0.9	0.59	0.125	1976	−12.2	7.19	0.089
COP Spatial Reasoning	2169	−1.15	0.97	0.237	1973	−4.12	12.2	0.734

Linear Mixed Model; (DCT marker) ∼ APOE/PRS + age + sex + education

## Discussion

The objective of this study was to examine DCTclock™ performance as a potential AD susceptibility biomarker in a large adult sample using the FHS. We performed association analyses of DCTclock™ composite scores with APOE ε4 status and our constructed AD polygenic risk score. The directionality of the effect sizes measures better or worse performance on the DCTclock™, where each numerical decrease in the effect size represents an equivalent decrease in the digital marker. Our results indicate that AD genetic risk is linked to poorer DCTclock™ performance in adults, including older adults without dementia.

DCTclock™ performance decreased with advancing age, lower education, and the presence of one or more copies of APOE ε4. To our knowledge, ours is the first study to demonstrate significant differences in clock drawing performance on the basis of APOE status or PRS. Interestingly, women tended to perform better than men across most composite scores. Studies examining traditional clock drawing performance have generally found significant effects of age and education, but not sex, on performance (57). However, we recently reported better performance on DCTclock™ composite scores in women compared to men in a different sample (19). It should therefore be considered possible that the more in-depth capture of neuropsychological behavior with DCTclock™ may improve sensitivity to detect sex differences in clock drawing performance.

In our main analysis, we found that the DCTclock™ Total Score and composite scores Information Processing Speed (both command and copy conditions) and Drawing Efficiency (command condition) were most strongly associated with PRS and APOE. This finding is consistent with previous literature show that DCTclock™ Total Score is sensitive to AD-related neuropathology and neurodegeneration (58). The Information Processing Speed and Drawing Efficiency composites are closely related, together capturing latencies or pauses, time spent ‘thinking’ (i.e., not producing ink on the test page) versus ‘drawing’, and total drawing time adjusted for drawing size, ink use, and number of pen strokes. Andersen and colleagues have recently shown that non-motor ‘think’ time is associated with worse performance on episodic memory tests (59). We have recently found Information Processing Speed and Drawing Efficiency composite scores, in addition to the DCTclock™ Total Score, to be useful in differentiating MCI from normal cognition (19). Our findings suggest that these processing speed-related markers could reveal some of the earliest aspects of cognitive performance affected by early-stage AD (“prodromal hypothesis”), or alternatively represent points of cognitive variation that exist on the basis of genetic risk factors for AD across the adult lifespan (”phenotype hypothesis”) (60). Repeating our analysis in a younger cohort or obtaining other AD biomarker data for the sample could potentially help with making this distinction in future research (61).

Prior studies examining differences in cognitive performance on the basis of APOE status in healthy adults have shown that APOE carriers may show subtle differences in memory performance relative to non-carriers; however, relatively few studies have examined differences in other cognitive domains, such as processing speed, and results have been mixed (61, 62). More recently, studies have examined associations between PRS and cognitive trajectories in cognitively unimpaired and preclinical AD samples, with results showing that while PRS predicts cognitive decline, these effects are often primarily driven by APOE genotype (63, 64). We have found a similar pattern in this large-sample cross sectional analysis, and our findings further contribute to the literature by showing that the use of digital clock drawing and machine learning-derived scoring algorithms can identify nuanced performance inefficiencies to provide a novel susceptibility marker of AD. A future direction for this research will be to examine APOE and PRS in association with cognitive change in longitudinal dCDT data from the FHS, when available.

There were a number of sex-specific findings in our results. As mentioned above, women tended to perform better across most DCTclock™ composite scores. However, our sex-stratified APOE and PRS associations revealed similarly strong associations between APOE or PRS and DCTclock™ performance in men and women. Previous studies generally show that APOE is more likely to modulate risk for neurodegeneration in women resulting in faster cognitive decline in the presence of amyloidosis (65–67). However, the higher risk for Aβ deposition and cognitive decline in female APOE ε4 carriers also appears to be attenuated with advancing age (66). Therefore, it may be the case that the mean age (M=72.4) of women in our sample is above the age range where APOE ε4 potentially exerts the most effect on cognition.

### Strengths and limitations

The present study contributes to the extant literature validating the DCTclock™ as a cognitive screening tool that is not only sensitive to MCI subtypes and AD- related neuropathology, but is also useful as a digital biomarker of AD susceptibility due to its associations with AD PRS in older adults without dementia (19, 58). A strength of this study is its use of a large sample of community-dwelling older adults from the FHS, which represents the largest dCDT dataset available to date, and improves the strength and generalizability of our findings.

The clock drawing task is a longstanding and widely used neuropsychological tool that has become an essential part of dementia screening in Western countries (68, 69). The DCTclock™ measures aspects of clock drawing performance not captured with standard paper-pencil administration and utilizes automated scoring to improve the accuracy and reliability of measurement (70). Validation of the digitized version of the clock drawing task can therefore facilitate the transition from paper-and-pencil based administration to a more comprehensive, efficient, and sensitive screening tool.

Limitations of the clock drawing task include its inapplicability in some non-Western countries and potentially limited appropriateness for future generations of older adults who may be less familiar with analog clocks (71). A possible limitation of our study method itself is the use of composite measures. We decided to use the nine DCTclock™ generated composite measures, given that these summary-level scores are the most likely to be used clinically. We did not deviate from this plan, other than adding more runs with variant inputs. It is possible that if we worked with the broader array of process-related sub-scores generated by DCTclock™ we may have obtained different results. There could also have been an alternative way to construct the PRS, as there are multiple ways of doing so and continued active research in that area (72). Also in this study, race and ethnicity were not analyzed as a covariate; this was due to the low percentage of non-white individuals in FHS datasets who underwent DCTclock™ testing. A future study of FHS data could incorporate race and ethnicity once more digital clock drawing data has been collected from the younger and more racially and ethnically diverse generations of the cohort.

Finally, our findings were attenuated after excluding a small subset of participants with dementia, suggesting that this group contributed significantly to the cognitive phenotypic variability and may have driven some of the moderate effects that we initially observed. Although no significant associations remained between APOE and DCTclock™ variables after individual with dementia were excluded, two of the three significant associations between PRS and DCTclock™ performance persisted. This suggests that PRS uniquely identifies aspects of cognitive performance measured by DCTclock™ (information processing and drawing efficiency) that may have prognostic utility even among cognitively healthy older adults. This is a completely novel and important finding, particularly as the field of AD research seeks to identify screening tools that are scalable, efficient to administer, and sensitive to preclinical and prodromal AD.

### Future Directions

Although the findings of this paper provide support for the continued neuropsychological use of DCTclock™, more work must be done before DCTclock™ can be fully integrated as a screening tool in clinical settings, such as primary care. Findings from additional aspects of a large-scale DCTclock™ validation study using the FHS are forthcoming. Further research is needed to validate DCTclock™ in a diverse, real-world clinical setting and ultimately integrate DCTclock™ into electronic medical record (EMR) systems.

Additional future research is needed on genetic risk, both related to APOE (e.g., two APOE ε4 alleles [ε4/ε4] compared to [ε3/ε4]) and additional AD genetic markers. More research is also warranted to better understand sex-specific associations between cognitive performance on DCTclock™ and AD genetic risk. Future research should consider examining polymorphisms in the serpin family B member 1 (SERPINB1), which have been closely linked with AD-specific neuropathology in women only (73).

### Electronic Supplementary Material

Supplementary material, approximately 19.4 KB.
