# Supplementary material for: Digital Clock Drawing as an Alzheimer’s Disease Susceptibility Biomarker: Associations with Genetic Risk Score and APOE in Older Adults
Source: J Prev Alzheimers Dis. 2023 Apr 28;11(1):79–87. doi: 10.14283/jpad.2023.48 (PMC10794851; doi:10.14283/jpad.2023.48)
Supplement: Supplementary file 1 — Supplementary material, approximately 19.4 KB. [file 42414_2023_48_MOESM1_ESM.docx]

**Supplemental Table 1**. Nine composite measures generated by the DCTclock^TM^ digital clock test scoring algorithm.

| **Total Score** | A single score between 0 and 100 that captures overall performance across command and copy conditions |
| --- | --- |
| **Composite Scales:** |  |
| Drawing Efficiency | The efficiency the participant demonstrated during the process of drawing the clock. This considers metrics such as total time spent compared to amount of ink used, pen strokes and ink length, size of the drawing, etc. |
| Simple/ Complex Motor Operations | The motor components involved during the process of drawing the clock. This considers metrics including speed and oscillatory motion and can be helpful in parsing out graphomotor concerns. |
| Information Processing Speed | The ability to process information demonstrated during the process of drawing the clock. This considers metrics including latencies, pauses, and relative time spent thinking (without pen to paper) versus actively drawing. |
| Spatial Reasoning | The spatial abilities demonstrated during the process of drawing the clock. This considers metrics including geometric and spatial placement of the various properties of the drawing. |
| ***Note*:** Score calculation is automated and cloud-based. Composite and subscale scores are calculated for both command (COM) and copy (COP) conditions and normed with respect to cognitively healthy individuals. Composite scales are adjusted for age. | |

**Supplemental Table 2**. Number of data points from each cohort used in this study.

| Original Cohort (Generation 1) | 1 |
| --- | --- |
| Offspring Cohort (Generation 2) | 1809 |
| New Offspring Spouse Cohort | 37 |
| Generation 3 Cohort | 370 |
| Omni 1 Cohort | 142 |
| Omni 2 Cohort | 39 |
| Total *n* | 2398 |

**Supplemental Table 3**. Univariate association analyses of the nine composite DCTclock^TM^ measures with sex.

| **Sex** | | | | |
| --- | --- | --- | --- | --- |
| Variable | *n* | Beta | SE | p-value |
| DCT Total Score | 2339 | 2.39 | 0.90 | 0.014 |
| COM Drawing Efficiency | 2395 | 0.86 | 0.49 | 0.081 |
| COM Simple Motor | 2379 | 2.29 | 0.37 | < 0.001 |
| COM Information Processing | 2395 | 0.03 | 0.44 | 0.949 |
| COM Spatial Reasoning | 2384 | 0.78 | 0.81 | 0.335 |
| COP Drawing Efficiency | 2361 | -0.07 | 0.40 | 0.861 |
| COP Simple Motor | 2355 | 1.18 | 0.29 | < 0.001 |
| COP Information Processing | 2361 | 1.01 | 0.50 | 0.044 |
| COP Spatial Reasoning | 2355 | 0.63 | 0.80 | 0.430 |
|  | | | | |
| Linear Mixed Model | (DCT marker) ~ sex | | | |

**Supplemental Table 4**. Univariate association analyses of the nine composite DCTclock^TM^ measures with education.

| **Education** | | | | |
| --- | --- | --- | --- | --- |
| Variable | *n* | Beta | SE | p-value |
| DCT Total Score | 2323 | 5.37 | 0.58 | < 0.001 |
| COM Drawing Efficiency | 2351 | 2.55 | 0.29 | < 0.001 |
| COM Simple Motor | 2335 | 1.22 | 0.22 | < 0.001 |
| COM Information Processing | 2351 | 1.90 | 0.26 | < 0.001 |
| COM Spatial Reasoning | 2340 | 3.66 | 0.49 | < 0.001 |
| COP Drawing Efficiency | 2345 | 1.91 | 0.24 | < 0.001 |
| COP Simple Motor | 2339 | 0.77 | 0.18 | < 0.001 |
| COP Information Processing | 2345 | 2.08 | 0.30 | < 0.001 |
| COP Spatial Reasoning | 2339 | 2.41 | 0.48 | < 0.001 |
|  | | | | |
| Linear Mixed Model | (DCT marker) ~ education | | | |

**Supplemental Table 5**. Univariate association analyses of the nine composite DCTclock^TM^ measures with age.

| **Age** | | | | |
| --- | --- | --- | --- | --- |
| Variable | *n* | Beta | SE | p-value |
| DCT Total Score | 2339 | -1.16 | 0.06 | < 0.001 |
| COM Drawing Efficiency | 2395 | -0.41 | 0.03 | < 0.001 |
| COM Simple Motor | 2379 | -0.29 | 0.02 | < 0.001 |
| COM Information Processing | 2395 | -0.35 | 0.03 | < 0.001 |
| COM Spatial Reasoning | 2384 | -0.76 | 0.05 | < 0.001 |
| COP Drawing Efficiency | 2361 | -0.37 | 0.02 | < 0.001 |
| COP Simple Motor | 2355 | -0.24 | 0.02 | < 0.001 |
| COP Information Processing | 2361 | -0.48 | 0.03 | < 0.001 |
| COP Spatial Reasoning | 2355 | -0.59 | 0.05 | < 0.001 |
|  | | | | |
| Linear Mixed Model | (DCT marker) ~ age | | | |
